# Telomere- and oxidative stress dynamics in Psittacidae species with different longevity trajectories

**DOI:** 10.1007/s11357-024-01397-5

**Published:** 2024-10-25

**Authors:** Angélica Domínguez-de-Barros, Inés Sifaoui, Roberto Dorta-Guerra, Jacob Lorenzo-Morales, Rafael Castro-Fuentes, Elizabeth Córdoba-Lanús

**Affiliations:** 1 Instituto Universitario de Enfermedades Tropicales y Salud Pública de Canarias (IUETSPC), Universidad de La Laguna, La Laguna, Tenerife Spain; 2 Departamento de Matemáticas, Estadística e Investigación Operativa, Facultad de Ciencias, Sección de Matemáticas, Universidad de La Laguna, La Laguna, Tenerife Spain; 3 Departamento de Obstetricia y Ginecología, Pediatría, Medicina Preventiva y Salud Pública, Toxicología, Medicina Legal y Forense y Parasitología, Facultad de Ciencias de la Salud, Sección Medicina, Universidad de La Laguna, La Laguna, Tenerife Spain; 4 Departamento de Ciencias Médicas Básicas, Facultad de Ciencias de la Salud, Sección Medicina, Universidad de La Laguna, La Laguna, Tenerife Spain; 5 Centro de Investigación Biomédica en Red de Enfermedades Infecciosas (CIBERINFEC), Instituto de Salud Carlos III, 28029 Madrid, Spain

**Keywords:** Aging, Longevity, Psittacidae, Telomere dynamics, Oxidative stress, Breeding

## Abstract

Telomeres, conserved DNA sequences at chromosome ends, naturally shorten with age, exacerbated by external factors like environmental challenges and reproduction. Birds, particularly psittacine, are gaining prominence as new aging models over the years because of their unique characteristics. This study explores erythrocyte telomere length (TL) and oxidative stress markers in plasma of long- and short-lived captive birds of the order Psittaciformes over four years. Long-lived birds consistently exhibited longer TL than short-lived ones (*p* = 0.012) but experienced a more pronounced TL shortening rate (*p* < 0.001) than short-lived ones. Breeding individuals experienced increased TL shortening compared to non-reproductive counterparts in long-lived birds (*p* = 0.008). Interestingly, long-lived birds showed a higher total antioxidant capacity than short-lived ones (*p* < 0.001), which was also increased during breeding (*p* = 0.026). A significant correlation was found between the telomere length shortening rate within the 4 years of study and the accumulated oxidative stress (*r* = 0.426, *p* = 0.069) in short-lived birds. These findings shed light on TL and oxidative stress dynamics over time, revealing distinct patterns influenced by life-traits among longevity groups.

**Supplementary Information:**

The online version contains supplementary material available at 10.1007/s11357-024-01397-5.

## Introduction

Biological aging is defined as a progressive loss of functions after the organism reaches maturity, eventually leading to its death [1]. Plenty of research has been carried out over the last years in the field on the mechanisms leading the aging process [2, 3]. Telomeres that stand within the molecular mechanisms of aging are highly conserved non-coding DNA sequences (TTAGGG)_n_ at the end of the chromosomes of vertebrates [4]. Among its functions are protecting the genome from degradation and interchromosomal fusion. A continuous and natural shortening of the telomere sequence occurs after each cell division due to the “end-replication problem” [5]. But telomeres are a dynamic structure that can also be lengthened over time depending on different mechanisms [6]. In the absence of repairing mechanisms, telomeres can shorten to a critical length, triggering mechanisms of replicative senescence or apoptotic cell death.

Telomere length varies among species and within organisms of the same species, it even can differ between different habitats [7]. Initial telomere length may be determined by heritage and genetic factors [8], but as they shorten with age, this can be aggravated due to both intrinsic and extrinsic processes, such as environmental challenges [9], or the reproductive effort [10, 11]. It is noteworthy in relation to reproduction, inflammation and/or oxidative stress as the most common processes involved [12].

Telomere length has been shown to predict longevity, however not only telomere length, but its dynamic, can settle a better estimate of life expectancy, as referenced by others [13]. Still, less is clear about how the different life-histories in bird populations, longevity, disease, and other processes take part in telomere dynamics [2, 14].

For the study of telomere length and its dynamics, mice (*Mus musculus*) have been used as the standard animal model for mammals, and additionally, other animal models have emerged for different species over the years, for example, the domestic chicken *(Gallus gallus domesticus*) has been used for birds [15]. Even so, there is a need to find other aging models that are more suitable for telomere research relative to the human aging process. In this case, birds have gained prominence as possible animal models in the last decades [15, 16], since, despite their physiological characteristics, (elevated corporal temperature, higher metabolism and O_2_ partial pressure than mammals… etc.), that suggest that they are more predisposed to suffer oxidative damage, some of them manage to live 2–3 times longer and age slower than other mammals of the same size. Among birds, psittacine are a group of birds with exceptional life expectancy, probably as a product of key evolutionary traits in terms of cognition and social skills (delayed reproduction, heavy investment in reduced offspring, large brains, vocal communication, and social information transfer) which reduce extrinsic mortality and thus increase longevity [17]. The order Psittaciformes includes diverse families from South America, Asia, and recently incorporated from New Guinea, including macaws, parrots, and related forms (parakeets).

Although it is very difficult to perform longitudinal studies under natural conditions, studies up to date have reported that telomeres shorten over the years in birds, and even differences in this shortening ratio according to the longevity of these birds [13, 18, 19]. Nevertheless, scarce information exists relative to the rate of telomere shortening within Psittaciformes, and the present study may be one of the first to compare this phenomenon in parrots of different well-known ages and different longevity strategies within the same order. In addition, the birds under study belonged to the Loro Parque Fundación, Tenerife, Spain, which holds the largest genetic reserve of parrots, so it guarantees that they are preserved in perfect health and without the influence of external factors such as predation, diet, or extreme environmental factors. This control helps the researchers isolate specific variables, understanding how these biomarkers respond to the different longevity strategies, and improving knowledge of the fundamental processes underlying aging in this area.

This study is the continuation of a previous cross-sectional research studying telomere length in two psittacine longevity groups [11], where larger telomeres were found in long-lived birds. An impact on reproduction was also verified, showing that the individuals that had bred had shorter telomeres than non-reproductive ones.

The aim of the present study is to determine telomere length dynamics and oxidative stress levels in birds of the order Psittaciformes with different longevity (long- and short-lifespans) followed over four years. Firstly, to study its telomere dynamics throughout the years according to the bird’s longevity. Secondly, to establish the effect of oxidative stress on telomere length, especially when facing the breeding process.

## Methods

### Selection of individuals

Psittacine birds of different species and longevities (long- and short-lived) were analyzed annually over the course of four years (2019–2022). The birds were ringed and sex was determined by a video endoscopy (Storz, RP100, and optics Hopkins 30º 2.7 mm/18 cm) performed by an expert veterinary team. This data was registered at the government entities (CITES, https://cites.org/esp). The study consisted of three time-points of sampling: the first one corresponded to the baseline moment of the study (t_0_), the second (t_1_) was performed after two years, and the third (t_2_) to the last year of sampling. The criteria for choosing the species for this study are based on a database/literature search on maximum longevity in the Psittacidae family and already detailed in a previous study of our group [11]: similarity in body size, similar age at sexual maturation, diet administered, and sample range available was considered (Table 1 and Supplementary Table 1). Information about these parameters was registered at each sampling time-point. In total, 202 biological samples were taken during the whole follow-up of the cohort. Sample sizes declined over the years (from 81 to 57 individuals, Table 1) due to the mortality of the monitored birds or their withdrawal to the veterinary clinic in case of any infection, therefore, they were excluded from further study to fulfil the expectations of an observational study of animals in good health and conservation status.

**Table 1:** General information about the long-lived and short-lived birds of the order Psittaciformes selected in the three-time sampling points study

Group	Species	N^a^			Age (yrs)^b^	Max Lifespan (yrs)^c^	Body mass^d^ (g)	Breeding age^e^ (yrs)
		n_0_	n_2_	n_3_				
**Long-lived birds**	*Amazona barbadensis*	22	20	19	13 (3–29)	35	319.3 ± 50.88	5
	*Anodorhynchus hyacinthinus*	8	4	3	29 (23–33)	38	1477.7 ± 117.04	10
	*Cacatua moluccensis*	9	9	7	25 (9–35)	65	877.8 ± 112.75	10
	*Ara macao*	8	8	8	20 (6–36)	33	998.0 ± 115.18	10
**Short-lived** **birds**	*Agapornis taranta*	8	3	3	7 (1–7)	15	47.6 ± 2.43	2
	*Psitteuteles* *goldiei*	19	15	12	1 (1–7)	10	51.8 ± 5.26	2
	*Trichoglossus johnstoniae*	7	5	5	7 (2–9)	17	55.9 ± 3.80	3
	Total	81	64	57				

^a^N_i_: number of birds corresponding to the time-points of sampling [2019(n_0_), 2021(n_2_), 2022(n_3_)]

^b^Age: median (P_25_-P_75_) in years (yrs)

^c^Maximum Life expectancy data and breed information of the Psittacidae birds recorded from AnAge database (https://genomics.senescence.info/species/index.html) and ZIMS Species 360 Global Information Serving Conservation database (https://zims.species360.org/Login.aspx?ReturnUrl=%2f)

^d^Average body mass of the birds during the four years of study (g) (mean ± standard deviation (S.D))

^e^Age at which the birds reach sexual maturity according to AnAge database (https://genomics.senescence.info/species/index.html) in years (yrs)

### Sample collection

The birds were placed in the breeding center of Loro Parque Fundación in separate galvanized wire mesh cages, differentiated by species, normally selected in breeding pairs or in groups of youngsters when they have not reached maturity or an assigned partner. At the moment of the different veterinary check-ups and blood withdrawals, the veterinary team captured the birds with a specific design net. Sampling consisted of blood withdrawal obtained from routine annual check-ups properly carried out by the veterinarian team, without any harm or risk to the life of the animals under study, according to the ARRIVE guidelines. The birds were immediately placed back in the same facility after the procedure. This process was done after the breeding season of each longevity group, so as not to increase stress situations. Short-lived birds were sampled in September and the long-lived birds in November. Blood was extracted from the right jugular vein of each bird, then they were centrifuged within the next 2 h at 3000 rpm for 10 min to separate plasma. At the same time of sample collection, other important parameters, such as body weight, were registered.

### Telomere length measurement

Relative telomere length (rTL) was measured as reported in previous research of our group [11]. Briefly, telomere length was assessed using the real-time quantitative PCR (qPCR) adapted to birds by Criscuolo et al. 2009 [20]. rTL is expressed as the T/S ratio, measuring the relative amount of telomeric repeats of the *Tel* gene (T), versus (vs) a single copy reference gene (S), which was *GAPDH,* and corrected by the method of Pfaffl, 2001 [21]. This ratio reflects relative inter and intra-individual differences in telomere length.

Real-time qPCR was performed in a StepOne Plus thermocycler (AppliedBiosystems, ThermoFisher Scientific, MA, USA) under the following conditions (Supplementary Table 2) as detailed previously [11]. In summary, the reaction included 200 nM of primers and 1.25 ng of DNA per reaction in a final volume of 10 µl, containing 5 µl of Power SYBR® Green PCR Master Mix (AppliedBiosystems. MA, USA). All samples were run in duplicate. A reference sample DNA belonging to the species *Anodorhynchus hyacinthinus* was used, from which a standard curve was created at the following concentrations: 40 ng/µl, 10 ng/µl, 2.5 ng/µl, and 0.6 ng/µl. The qPCR reactions showed a mean efficiency of E = 98.1% and correlation of R^2^ = 0.94 for telomeres, and E = 95% and R^2^ = 0.99 for *GAPDH* respectively. Intra-plate coefficients of variance (CV) were calculated between the replicates and samples with CV > 5% were excluded from further analysis. Inter-plate CV for the calibrator sample was calculated to be < 7%.

### Quantification of the total antioxidant capacity (TAC)

The measurement of antioxidant capacity in 10 µl of serum samples was performed considering the reduction of ferric ion (Fe^3+^) when combined with a radical such as TPTZ (tripyridyl-s-triazine), that result on ferrous ion (Fe^2+^). The determination of Fe^2+^ concentration was carried out by preparing a working solution containing Acetate Buffer (30 mM), Iron Trichloride (20 mM) and TPTZ (10 mM) in a 10:1:1 ratio. Each sample was evaluated in duplicate.

The reaction was measured by spectrophotometry and absorbance was quantified at 593 nm on the EnSpire Multimode Plate Reader (Perkin Elmer, Madrid, Spain). The quantification of equivalent units of Fe^2+^ (µM Trollox) was obtained through the Standard Curve of Trollox by using the Total Antioxidant Capacity Assay Kit (Abnova ™ GmbH, Taiwán).

### Quantification of lipid peroxidation products by the TBARS assay

Lipid peroxidation was determined through colorimetric detection method in serum samples, where the reaction of the main lipid oxidation product, malondialdehyde (MDA), with thiobarbituric acid (TBARS) was observed. The assay was carried out using the OxiSelect TBARS Assay Kit (Cell Biolabs, Inc, USA) following the instructions of the manufacturer. The MDA-TBA adduct formed was measured fluorometrically on the EnSpire Multimode Plate Reader (Perkin-Elmer, Madrid, Spain). Quantification of MDA (µM) was obtained through the Standard Curve. Each sample was evaluated in duplicate.

### Statistical analysis

Continuous variables were described using means and standard deviation or median and percentiles (P_25_-P_75_) when not normally distributed. Outliers in the data were assessed by inspection of boxplots, and, when necessary, normality distribution of variables was assessed by Shapiro–Wilk's test or Kolmogorov–Smirnov test as appropriate (*p* > 0.05). The homogeneity of variances was assessed by Levene's test for equality of variances. The variables of *Total Antioxidant Capacity* (TAC) and *Lipid peroxidation products* (thiobarbituric acid) (TBARS) were log-transformed prior to analysis.

Several mixed-models of repeated-measures, ANCOVA´s, were performed. The assumed covariance structure for these analyses was compound symmetry. This model uses all available data, providing more accurate estimations without the need to remove subjects from the analysis. All assumptions for general Linear Mixed Models were checked. Time-factors interactions were included in all models and when necessary, all pairwise comparisons were run for statistically significant simple main effects with reported 95% confidence intervals and p-values Bonferroni adjusted.

Several models were constructed to assess the impact of independent variables, including *longevity*, *time*, *species*, and *reproduction*, as fixed factors in the analysis of *Telomere Length* (TL), log-transformed *Total Antioxidant Capacity* (logTAC), and log-transformed *Lipid peroxidation products* (logTBARS) as response variables over a four-year follow-up period (three-sample points). Additionally, standardized age was incorporated as a covariate in the analysis of telomere length, since the passing of 1 year of age is not the same for the different species within each longevity strategy. The influence of sex was also examined in the model, but the results did not yield statistical significance, leading to its exclusion from further analysis.

The *telomere rate of change* was calculated as the difference between the relative telomere length at baseline (t_0_) and the last year of sampling (t_2_). The associations between telomere length change with the variables of oxidative stress were analyzed for each longevity group using Pearson’s correlation coefficients adjusted by age.

All statistical analyses were performed by using SPSS v.25.0 (IBM Statistics) and two-tailed p-values < 0.05 were considered significant. Graphs were designed with GraphPad Prism v9.0 (Dotmatics, GraphPad Software, San Diego, California, United States).

## Results

### Telomere length and changes over time

**Fig. 1:**
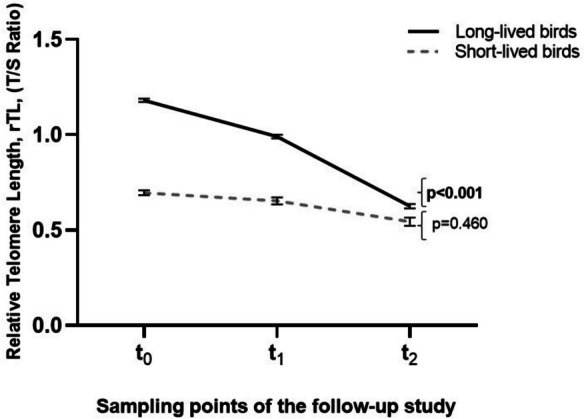
Relative Telomere Length (rTL) shortening of the psittacine birds included in this study during the follow-up (long- and short-lived birds)

The cross-sectional analysis revealed that long-lived birds had longer telomeres (rTL) when compared to short-lived birds during the study (Supplementary Fig. 1). Remarkably, when performing the longitudinal analysis, we discovered a significant difference in the telomere shortening rate over time depending on longevity (*p* = 0.012) (Fig. 1). Long-lived birds showed a greater shortening over time by 0.191 rTL units (CI 95%: 0.004—0.378; *p* = 0.043) from t_0_ to t_1_, by 0.365 rTL units (CI 95%: 0.162—0.569; *p* < 0.001) from t_1_ to t_2_, and by 0.556 rTL units (CI 95%: 0.357—0.755; *p* < 0.001) from t_0_ to t_2_. For short-lived birds, there were no differences in rTL over time (*p* = 0.355) (Fig. 1). We found a strong correlation between baseline telomere length and the rate of change in TL (*r* = 0.663, *p* < 0.001 for long-lived birds and *r* = 0.666, *p* = 0.002 for short-lived birds) (Supplementary Fig. 2).

**Fig. 2:**
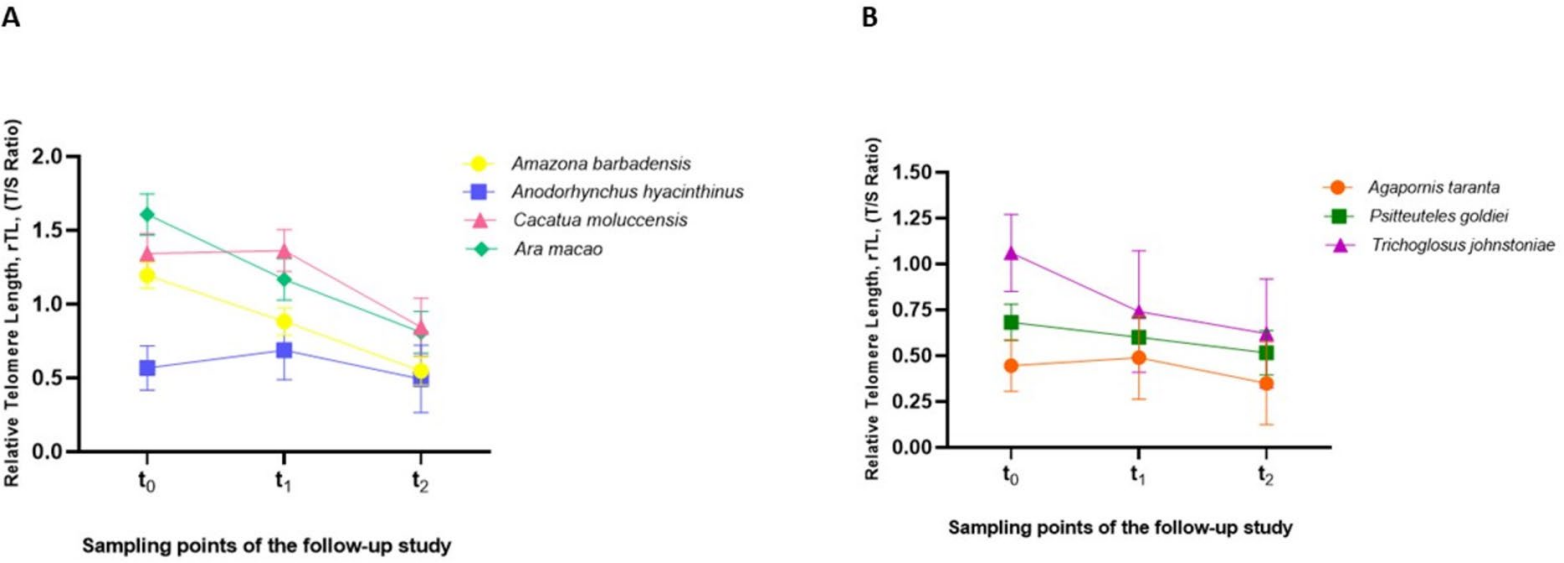
Relative telomere length (rTL) of the different species of long- and short-longevity birds included in this study. **A**) rTL dynamics of long-lived species during the 4-years follow-up. **B)** rTL dynamics of short-lived species throughout the years of study

The longitudinal analysis of rTL between the different species of both longevity groups revealed similar shortening of their telomeres over time (*p* = 0.162 and *p* = 0.986 for long- and short-longevity birds, respectively). In the long-lived birds' group, all species shortened their telomeres in like manner, although maintaining telomere length differences between them over time (*p* = 0.001). *Anodorhynchus hyacinthinus* had the shortest telomeres when compared to the other long-lived species, while *Ara macao and Catatua moluccensis* shared the largest telomeres within the group. *Ara macao* had similar telomere length to *Cacatua moluccensis* (*p* = 1.000) and to *Amazona barbadensis* (*p* = 0.064), but importantly had longer telomeres than *Anodorhynchus hyacinthinus* (*p* = 0.003) **(**Fig. 2a**)**. Within the short-lived birds, only *Trichoglossus johnstoniae* showed longer telomeres than *Agapornis taranta* (*p* = 0.046) (Fig. 2b).

To analyze the impact of reproduction on rTL, we grouped the long and short-longevity birds under study into two categories. The first category never underwent the reproductive process, while the second bred at least once in the follow-up. Entering the reproductive state had a significant impact on the rTL dynamics of the long-lived birds breeding individuals compared to non-breeding ones over time (*p* = 0.035). However, the magnitude of their rTL difference decreased during the study (Table 2). On the other hand, the rate of the rTL shortening in breeding individuals vs. non-breeding in the short-lived birds group was not significant over time (*p *= 0.854) (Table 2).

**Table 2:** rTL of long- and short-lived psittacine birds considered breeder vs. non-breeders at each sample point over the follow-up study

Group	Breeding	N^a^	rTL^b^			p-value^*^
Long-lived birds	Never bred ind	12	1.585 ± 0.16	1.113 ± 0.16	0.698 ± 0.18	*p* = 0.035
	Breeding ind^c^	35	1.041 ± 0.09	0.962 ± 0.09	0.609 ± 0.09	
	*p-value*^*d*^		*p* = 0.008	*p* = 0.447	*p* = 0.676	
Short-lived birds	Never bred ind	16	0.694 ± 0.14	0.621 ± 0.15	0.474 ± 0.17	*p* = 0.854
	Breeding ind^c^	18	0.675 ± 0.13	0.658 ± 0.13	0.567 ± 0.13	
	*p-value*^*d*^		*p* = 0.933	*p* = 0.875	*p* = 0.703	

^a^N_i_: number of birds categorized as if they underwent the reproductive process or not during the 4- year follow-up study

^b^rTL: Relative telomere length expressed as mean ± standard error (S.E.)

^c^Individuals that bred at least once within the four years of study

^d^GLMM Bonferroni post-hoc pair comparisons *p-values*

T_i_: Representation of this study sampling points (t_0_ = 2019, t_1_ = 2021, t_2_ = 2022)

^*^Significance level (*p* < 0.05)

### Markers of oxidative stress over time

**Fig. 3:**
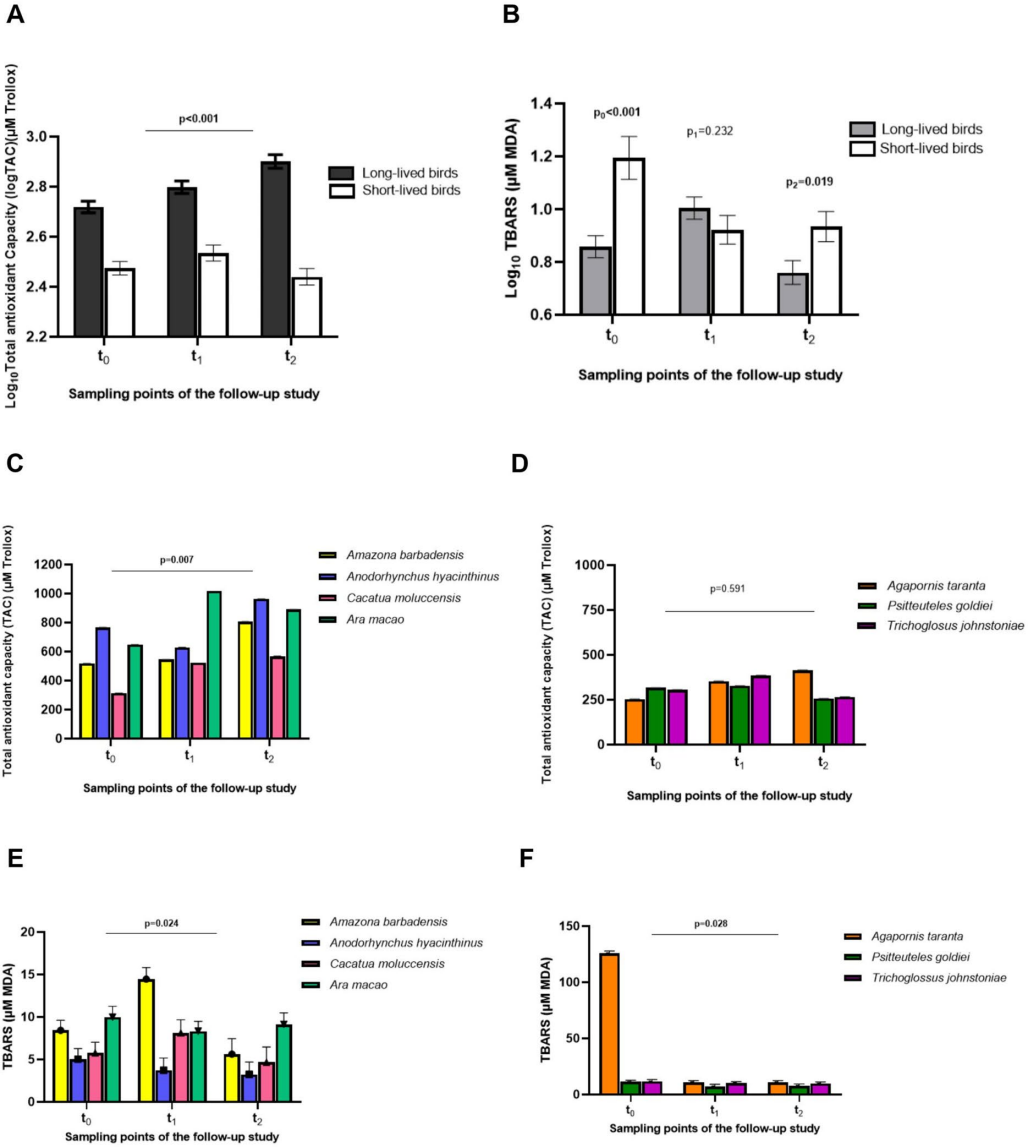
Total Antioxidant Capacity (TAC) and Lipid peroxidation products (TBARS) levels in the psittacine birds included in this study. **A)**. TAC levels of long- and short-lived birds over time. **B)** TBARS levels of long- and short-lived birds through the years of study. **C)** TAC levels of long-lived species at each sampling time-point. **D**) TAC levels of short-lived species at every sampling time-point. **E**) TBARS levels of long-lived species at each time-point of sampling. **F)** TBARS levels of short-lived species at every sampling time-point of this study

Long-lived birds had increased levels of total antioxidant capacity (TAC) than short-lived birds throughout the follow-up study (*p* < 0.001). Even, TAC of long-lived birds increased by 0.245 (CI95%: 0.175—0.315), 0.263 (CI95%: 0.184—0.343), and 0.461 (CI95%: 0.377—0.545) units with respect to the short-lived birds over the three sampling-points (*p* < 0.001 for t_0_, t_1_, t_2_, respectively) (Fig. 3a). On the contrary, short-lived birds accumulated more products of lipid peroxidation products (TBARS) than long-lived birds, which was evidenced significatively at t_0_ by 0.336 units (CI95%: 0.155—0.516; *p* < 0.001), and in t_2_ by 0.174 units (CI95%: 0.029—0.318; *p* = 0.019) (Fig. 3b). There was no interaction of sex with longevity for TAC and TBARS concentrations over the follow-up (*p* = 0.980 and *p* = 0.200, respectively). Interestingly, in short-lived birds, we found a significant correlation between those birds that shortened their telomeres and the oxidative stress accumulated at the last sampling point (t_2_) (*r* = 0.693, *p* = 0.026) (Supplementary Fig. 3).

The longitudinal analysis revealed that TAC levels varied within the different long-lived species among the follow-up (*p* = 0.007). *Ara macao* and *Anodorhynchus hyacinthinus* were the species that tended to have higher antioxidant capacity concentrations during follow-up, especially when compared to *Cacatua moluccensis* [*Ara* 648.63 ± 1.12 vs *Cacatua* 314.05 ± 1.12 µM of Trollox in t_0_ (*p* < 0.001); 1018.59 ± 1.12 vs 523.60 ± 1.12 µM of Trollox in t_1_ (*p* < 0.001); and 891.25 ± 1.12 vs 567.54 ± 1.18 µM of Trollox in t_2_ (*p* = 0.108)] (Fig. 3c), even though the differences in TAC levels between long-lived species became less significant over time. On the other hand, short-lived species were found to have similar TAC levels throughout the study (*p* = 0.591) (Fig. 3d). Long-lived species also were found to have significant differences in TBARS over time (*p* = 0.024) (Fig. 3e). In the short-lived birds' group, TBARS levels varied within the species (*p* = 0.028), especially in the first time-point of sampling, t_0_ [*Agapornis* 120.22 ± 1.67 vs *Psitteuteles* 12.02 ± 1.24 µM MDA (*p* < 0.001); vs *Trichoglossus* 12.88 ± 1.35 µM MDA (*p* = 0.001)] (Fig. 3f).

**Fig. 4:**
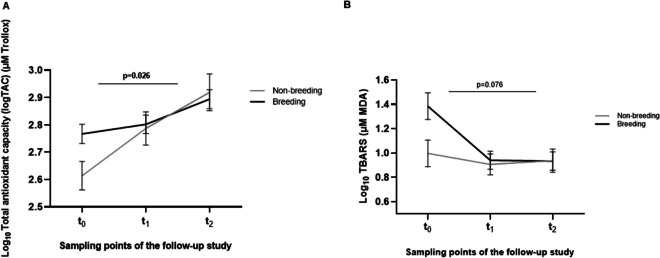
Biomarkers of stress in breeding vs non-breeding individuals of the different longevity psittacine birds included in the study. **A)** TAC levels of the long-lived birds’ group that had reproduced compared to non-reproductive individuals over time. **B)** TBARS levels of breeding individuals in the short-lived birds group compared to non-breeding over the four years of study

When examining the effects of reproduction on oxidative stress markers, we observed that breeding individuals of long-lived birds exhibited increased levels of TAC compared to those that had never reproduced over the follow-up (*p* = 0.038), this was evidenced, especially in t_0_, (*p* = 0.005). After that, the magnitude of the differences between breeders and non-breeders decreased over time (Fig. 4a). We observed the opposite effect in short-lived birds, without significant differences between breeders and non-breeding individuals [273.53 ± 1.07 vs 329.61 ± 1.07 µM of Trollox in t_0_ (*p* = 0.047); 363.08 ± 1.08 vs 322.11 ± 1.08 µM of Trollox in t_1_ (*p* = 0.278); and 295.80 ± 1.07 vs 244.34 ± 1.09 µM of Trollox in t_2_ (*p* = 0.104) in breeders vs non-breeders, respectively].

Regarding TBARS in the short-lived birds' group, individuals who had bred accumulated more lipid damage than those who had not reproduced the first year of study, but then the differences between each sample point decreased until they reached similar MDA concentrations (µM) over time (*p* = 0.076) [t_0_: 1.384 ± 0.11 breeders vs 0.996 ± 0.11 non-breeders (*p* = 0.027); t_1_: 0.940 ± 0.07 vs 0.906 ± 0.08 (*p* = 0.793); t_2_: 0.933 ± 0.075 vs 0.937 ± 0.09 (*p* = 0.974)] (Fig. 4b). On the other hand, reproduction did not show to have any effect on oxidative stress (TBARS levels) of long-lived birds (*p* = 0.305).

## Discussion

### Telomere length

In this work, an exhaustive monitoring of the telomere dynamics of different psittacine birds has been performed over four years. This study provides interesting findings regarding telomere attrition in the different species of the order Psittaciformes; long-lived birds have a longer initial telomere length compared to short-lived ones, which suggests an advantage for them in terms of longevity.

Telomere length has been described as a good candidate biomarker of biological aging that may provide valuable information of the biological state and be associated with markers of individual quality in humans and other animal species, such as mammals or birds [5, 22, 23]. In a first cross-sectional study over psittacine birds published by our group [11], relative telomere length (rTL) was found to be inversely correlated with age in both long-lived and short-lived birds. It has been reported that longevity and life expectancy are closely related to the rate of telomere erosion, with long-lived birds showing less telomere shortening [13, 18, 19].

So, although our results of greater shortening in long-lived birds differ from other studies previously mentioned, other studies have shown that telomeres may not shorten at a constant rate throughout life, despite previous assumptions [24]. Telomeres experience an accelerated shortening at the early stages of life, followed by a kind of stabilization phase in adulthood, which has led some cross-sectional studies to not reveal possible outcomes that only become apparent in the long run [6]. This could explain the differences we observed concerning the telomere length and between the biomarkers analyzed, which may have lacked significance when studied at certain stages of life. On the other hand, some studies have found that the telomere dynamics is not linear. In long-lived birds, an increase in telomere length has been observed [25]. Also, long telomeres were found to be better predictors of survival than shorter telomeres in studies conducted on long-lived birds [26]. Therefore, longitudinal studies are needed to achieve feasible and reliable results.

The present study is the first one that we are aware of that evaluates telomere dynamics in different species of psittacine birds. In concordance with our previous research, even though all long-lived species showed the same pattern of telomere shortening over time, the different species showed differences from each other with respect to their rTL. Species like *Amazona barbadensis* and *Ara macao*, despite their advanced age, still maintained longer telomeres compared to *Anodorhynchus hyacinthinus*. Lastly, the species of the long-lived group that had the longest telomere length also behave differently; while *Ara macao* experiences a gradual telomere shortening, *Cacatua moluccensis* appears to present oscillations in its telomere length over the follow-up. This can be explained by Pauliny et al. (2012), who reported that individual differences in the rate of telomere shortening, including increases and decreases, can be detected in adult birds [25].

Various factors may explain why telomeres of long-lived birds shortened faster than the short-lived birds. The most significant one relies upon genetics. While these birds may experience a higher rate of telomere shortening, their initial rtL, which has an important heritable component, is much greater, allowing its telomeres to be constrained for longer. Also, we have to consider repair mechanisms that could counteract and promote the survival of these long-lived birds, as has been reported in other studies [27]. Also, our findings of an association between telomere shortening rate and the oxidative stress markers studied may indicate that for short-lived birds, the impact of the damage caused by lipid peroxidation can be reflected in the shortening of telomeres; while for long-lived birds, other mechanisms may be orchestrated which require further investigation. Lastly, the conservation status of the birds included in this observational study must be considered, as the birds were kept in controlled environments, with no external factors that could have affected wild birds' life. This fact could have had a positive effect on the telomeric dynamics of the birds, particularly for short-lived ones, who are more vulnerable in the wild once erasing/reducing the likelihood of death by external factors like accidents, infectious diseases, and predation decreases, life expectancy should increase [16].

Another important contribution of this research is that we could confirm the effect of reproduction on the telomere length of these birds. There is consistent evidence that reproductive effort is related to telomere attrition (in concordance with other studies performed in birds) [24, 28]. Although our results in short-lived birds were not significant and we could not point to significant differences within rTL shortening between sexes (as stated by Bauch et al. 2020) [29], we observed a clear tendency of pronounced telomere shortening for all birds when facing the reproductive situation. This may indicate that telomeres are good candidates as markers of individual quality, for both long- and short-term costs, as a higher or lower rate of telomere shortening may, for example, reflect a better ability to achieve higher reproductive performance and entail survival [22, 30].

### Oxidative stress biomarkers

Oxidative stress is implicated in most processes and may be one of the major underlying causes of physiological senescence [31]. In our study, we measured stress markers in psittacine birds over time, including its negative (lipid peroxidation) as its positive (antioxidant capacity) effects. It is important to emphasize the need for longitudinal studies of these specific markers in an organism because, as has already been observed, cross-sectional comparisons can change and present great variability of associations with other life parameters as has been found in the literature.

One of the main reasons for the differences found in research focused on oxidative stress is the choice of the sample of study, since the results of some markers differ when studied in a specific tissue or at a systemic level. In the present study we have used plasma separated from whole blood, useful for the measurement of 8-OHdG, lipid peroxidation products, and protein carbonylation as biomarkers of oxidative stress and DNA damage. Furthermore, many of these measurements are accompanied by assays that measure antioxidant capacity. Although these markers are widely used in birds, the data obtained has been inconsistent, and inferences made at the level of a whole organism may not be accurate [32].

Our results exhibited that long-lived birds have higher levels of antioxidants in plasma than short-lived birds consistently over time. This could mean that long-lived birds generally suffer low levels of oxidative stress, in agreement with what was reported by Xia & Møller, (2018) [33], or due to a response mechanism to any suffered damage, that caused the increased activation of the antioxidant system [34].

Previous cross-sectional studies have investigated age-related variations in stress markers in birds with different results [35–37]. We did find a trend of increased antioxidant capacity over time in long-lived birds, in concordance with what has been found by Devevey et al. (2014) where an increase in antioxidant substances was observed in other long-lived birds [38].

The findings presented may suggest the existence of individual oxidative phenotypes. TAC behaved differently in the species of the long-lived birds, with special attention to *Ara macao* and *Anodorhynchus hyacinthinus*. As stated in Vágasi et al. 2016, these biomarkers were found to be species-specific and can be used to establish comparison between species [39], so further studies of the behavior of the antioxidant system of these long-lived birds could incorporate new information in the study of longevity.

Also, studies by Romero-Haro et al. (2023) have found that captive birds with low levels of specific antioxidant substances, such as glutathione, presented longer telomeres [40]. Even though we did not look into other enzymatic antioxidant substances, but rather a systemic output, long-lived birds still showed greater rtL than short-lived ones, which presented shorter telomeres. This is another strategy in which birds can promote their longevity. As for long-lived species, *Anodorhynchus* was the one that accumulated less concentrations of MDA than other species in its group, and it is the one that showed the greatest antioxidant capacity and lowest rTL over the follow-up. It is also interesting to note that within the long-lived species that showed greater antioxidant capacity, differences in their telomere length were observed. This could indicate that their life strategies are different, and their antioxidant defenses may be used for different purposes.

An open question remains over which mechanisms produce variation in antioxidant capacity among birds, as they can have genetic variation, differ in access to dietary antioxidants, different susceptibility to oxidative damage, or early ontogenetic processes caused by heritability effects [37].

Regarding the effect of reproduction on stress markers, our findings line up with other studies that show that long-lived birds experience an increase in their non-enzymatic antioxidant defenses when facing challenging physiological events such as reproduction. This could help them cope with stress and may improve their reproductive success, survival rates, and even protect their offspring [31, 39, 41]. Likewise, the opposite outcome is observed in short-lived birds. We observed a trend where breeding individuals with a shorter lifespan experienced greater oxidative damage to their lipids than non-reproducing ones. These results are in line with previous studies that found higher levels of oxidative damage in tissues of breeding birds with greater reproductive effort [41]. As already suggested, short-lived birds, under pressure of allocating their resources in favor of reproduction rather than self-maintenance, suffer more oxidative damage and this may cause negative effects on their life expectancy in the long-term [42, 43]. However, to establish a firmer conclusion about the effect of reproduction of birds on their survival, a more exhaustive study is necessary with a greater sample and an extended variety of oxidant markers [15], considering different approaches regarding the oxidative status-survival relationship.

To summarize, the patterns observed for telomere and oxidative stress dynamics in these birds are the result of the complex interplay of many factors and differ between species or individuals, especially in the aging process.

This may be one of the first studies performed on Psittacidae birds that mainly compare how the different longevity strategies within the same order of birds develop through the years. Different studies have performed comparisons regarding telomere length, but these involved different animal families or classes [44]. Psittacidae birds differ in terms of age and longevity, ranging from approximately a decade to more than six, so this study is an important opportunity to examine telomere length and possible pathways underlying these markers under controlled conditions, providing valuable insights into the mechanisms of aging in these conditions. It is important to highlight that we have also focused on describing telomere dynamics in short-lived species since references about their telomere length are scarce in the literature [19].

Through working with captive parrots, particularly with the conservation efforts of Loro Parque Fundación, we were able to gather both quantitative and qualitative data over time, enabling us to conduct a longitudinal study and develop interventions to promote healthy aging and extend lifespan in both birds and other vertebrate species, including humans. By measuring telomere length (rTL) from whole blood and stress biomarkers from serum, we were able to work with a non-invasive method. The measurements of telomere length in blood are representative of what can be found in other somatic tissues [45]. A large number of species from the Psittaciformes order are classified as vulnerable to the threat of extinction according to the red list of the Congress of the International Union for Conservation of Nature (IUCN) in 2008 [46]. This reflects the importance for conservation centers to seek possible research lines focused on bioindicators of life expectancy and the biological status of these birds, since they are fundamental in the study of ecology and for the long-term conservation of endangered species.

This study has limitations as follows: Firstly, during the time of sampling, some birds may have been unavailable due to being in the clinic for check-ups or may have passed away due to their old age. Regarding that matter, to confirm our results we require a broader sample size and a longer period of measurements. Although other long-term longitudinal studies of avian species usually took around six to ten years, and some of them only measured rTL twice in the individuals' life [10, 13, 22, 26], we are aware that four-year follow-up study may be scarce, especially regarding the lifespan of the species included in this study. Secondly, our study has been conducted on captive birds. Therefore, there is a need to perform studies on parrots in wild conditions to test if our results correspond to what is found in nature. Psittacidae birds have been little studied in controlled environments, which makes them excellent study models of possible important markers in the study of aging [47]. We are confident that the present findings represent a starting point in psittacine birds' telomere dynamics studies.

In conclusion, our findings showed that telomere length in Psittacidae birds decreases over time. Interestingly long-lived birds experienced greater telomere shortening than short-lived ones but also presented an initial longer telomere length which could have genetic implications. Telomere length varied among the different long-lived species, which showed the different strategies and life traits adopted by each species to improve their survival. Reproduction has a noticeable impact on telomere length of both longevity groups. However, long-lived birds respond differently to this event. Long-lived birds showed an improvement in their antioxidant capacity, while short-lived birds exhibited more oxidative damage when facing the reproductive events over time. Further research on telomere variation and repair mechanisms over entire life spans with comprehensive, long-term ecological and genetic data are required.

## Supplementary Information

Below is the link to the electronic supplementary material.

Supplementary file1 (DOCX 3289 KB)

Supplementary file2 (XLSX 38 KB)

Supplementary file3 (DOCX 19 KB)

## Data Availability

The original contributions presented in the study are included in the article/supplementary material. Any additional inquiries can be directed to the corresponding author.

## Materials availability

This study did not generate new unique reagents.
