# Supplementary material for: Telomere- and oxidative stress dynamics in Psittacidae species with different longevity trajectories
Source: GeroScience. 2024 Oct 25;47(1):121–34. doi: 10.1007/s11357-024-01397-5 (PMC11872948; doi:10.1007/s11357-024-01397-5)
Supplement: Supplementary file 1 — Supplementary file1 (DOCX 3289 KB) [file 11357_2024_1397_MOESM1_ESM.docx]

**Supplementary figures
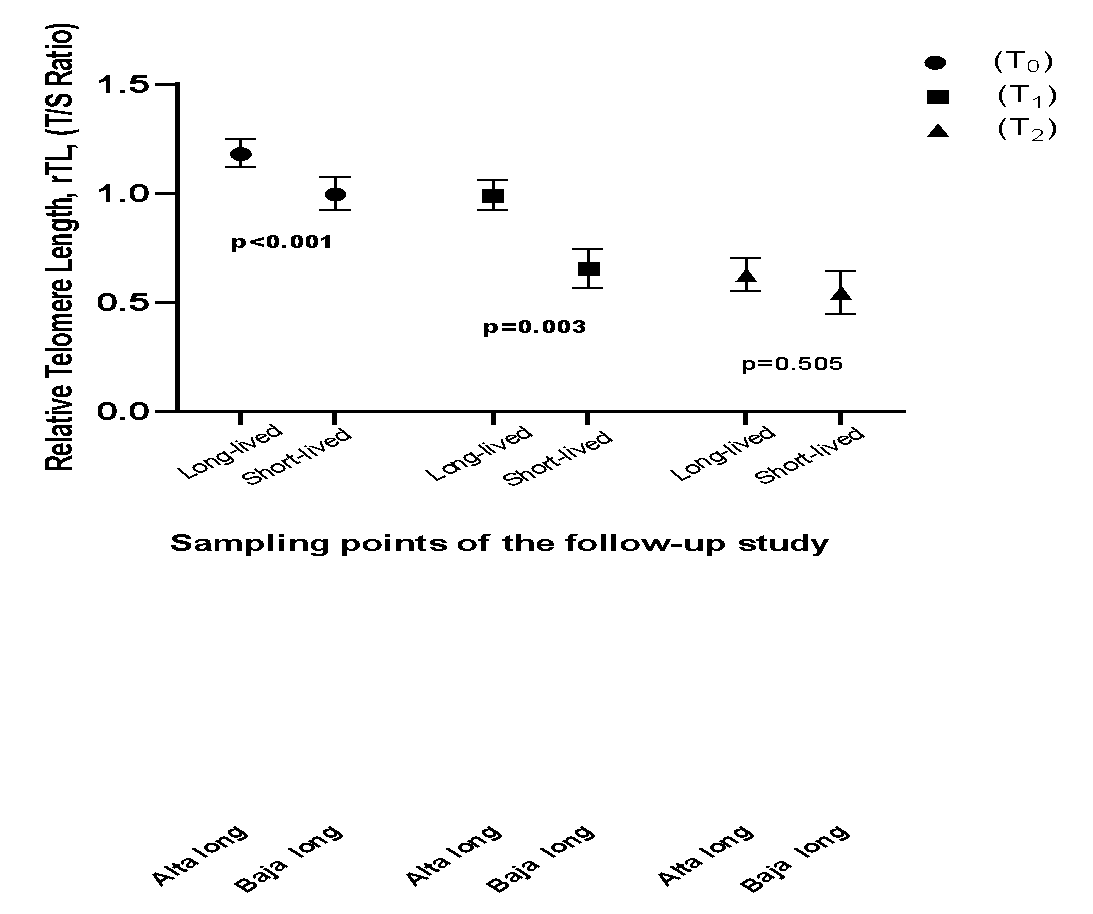
.**

**Supplementary Figure 1**. Relative telomere length (rTL) of long- and short-longevity birds in each time point (t_0_, t_1_, t_2_) over the 4-years of study.


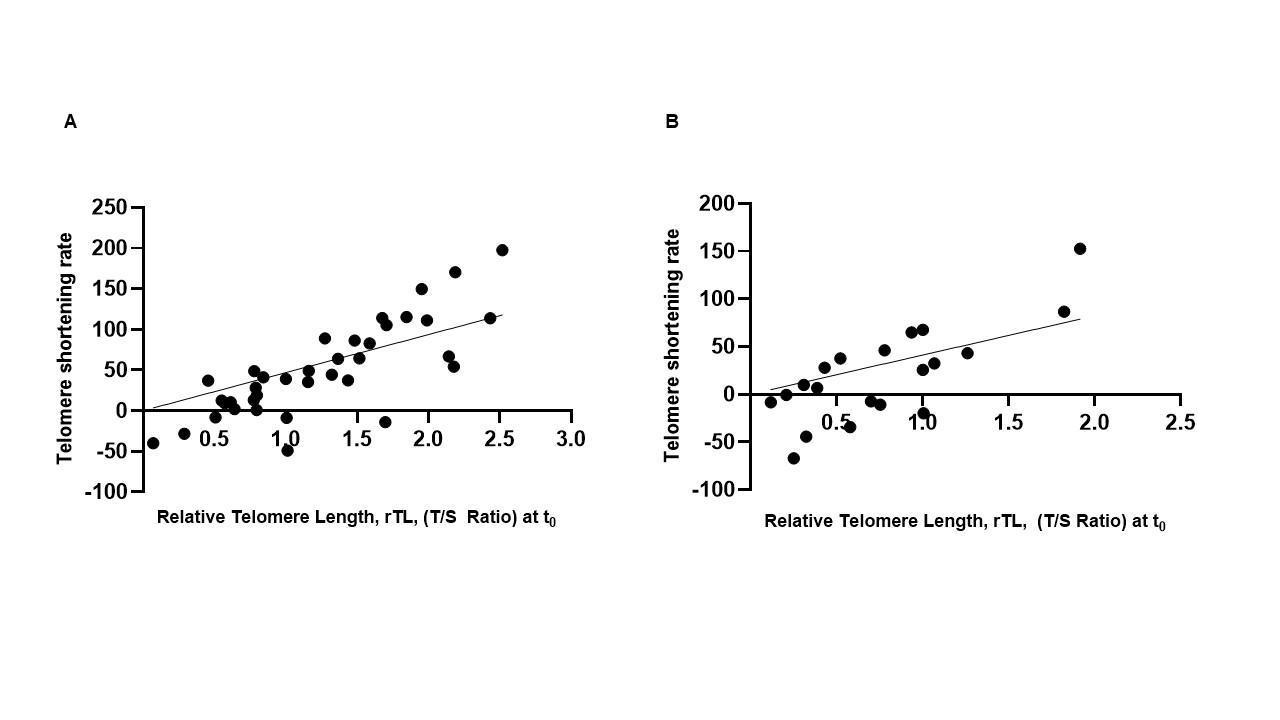


**Supplementary Figure 2**. Correlation between baseline telomere length and telomere shortening rate of change (%). **A**) In long-lived birds. **B)** In short-lived birds.


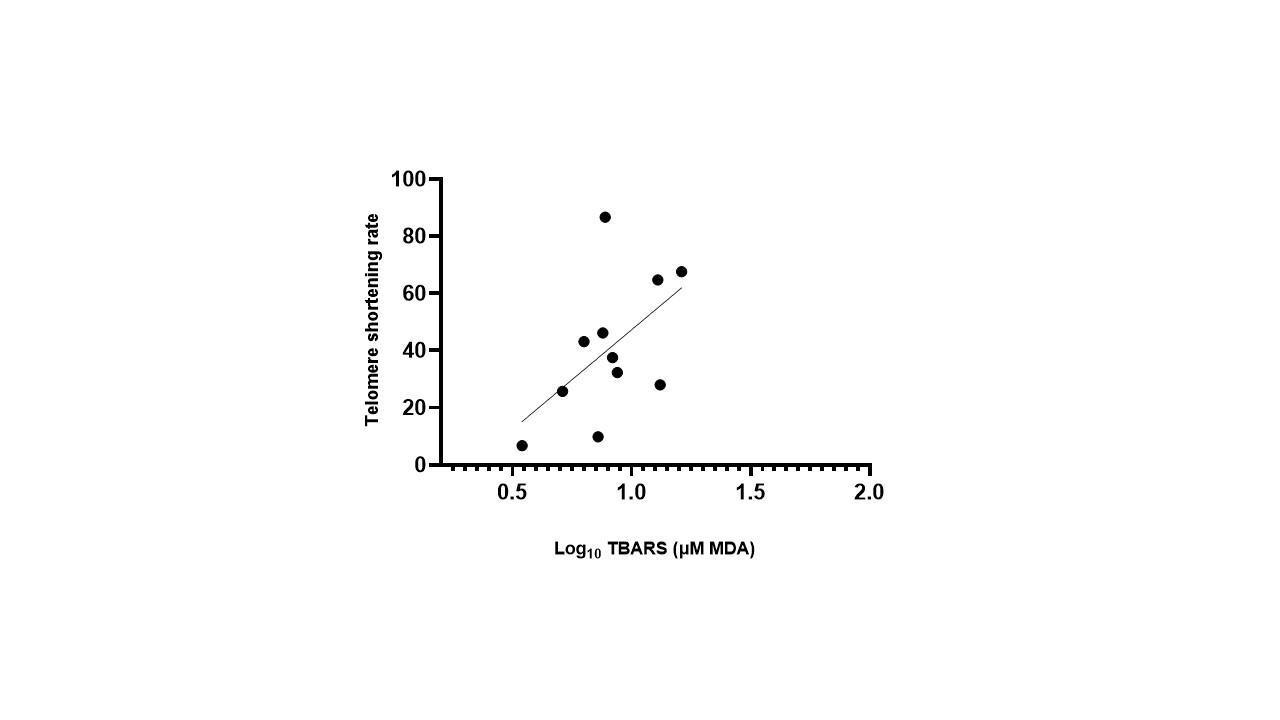


**Supplementary Figure 3**. Correlation between lipid peroxidation products (TBARS) and the telomere shortening rate change (%) in short-lived birds (including only individuals who shortened their telomeres during follow-up).
