# Supplementary material for: Telomere- and oxidative stress dynamics in Psittacidae species with different longevity trajectories
Source: GeroScience. 2024 Oct 25;47(1):121–34. doi: 10.1007/s11357-024-01397-5 (PMC11872948; doi:10.1007/s11357-024-01397-5)
Supplement: Supplementary file 3 — Supplementary file3 (DOCX 19 KB) [file 11357_2024_1397_MOESM3_ESM.docx]

**Supplementary table 2**. Specific sequence for each primer and qPCR amplification conditions to determine rTL of birds under study.

| **Sequences** | **PCR conditions** | |
| --- | --- | --- |
| Telomeres (*Tel*) | 95ºC 10 min  95ºC 15 sec  56ºC 30 sec  72ºC 30sec | 27x cycles |
| *Tel1b* Fw-  5’ CGGTTTGTTTGGGTTTGGGTTTGGGTTTGGGGTTTGGGTT-3’  *Tel2b* Rv-  5’ GGCTTGCCTTACCCTTACCCTTACCCTTACCCTTACCCT-3’ |  |  |
| Glyceraldehyde-3-phosphate dehydrogenase (*GAPDH*) | 95ºC 10 min  95ºC 15 sec  56ºC 30 sec  72ºC 30sec | 40x cycles |
| *GAPDH* Fw-  5’ GTGGTGCTAAGCGTGTTATCATC-3’  *GAPDH* Rv-  5` GGCAGCACCTCTGCCATC-3’ |  |  |
